# Plasma Exosomal miRNA-139-3p is a Novel Biomarker of Colorectal Cancer

**DOI:** 10.7150/jca.45548

**Published:** 2020-06-15

**Authors:** Wanchao Liu, Dianyu Yang, Longmei Chen, Qingqing Liu, Wenhui Wang, Zhenghua Yang, Anquan Shang, Wenqiang Quan, Dong Li

**Affiliations:** 1 Department of Clinical Laboratory, Baoshan District Hospital of Integrated Traditional Chinese and Western Medicine, Shanghai, 201999, China.; 2 Department of Clinical Laboratory, Shanghai Tongji Hospital, Tongji University School of Medicine, Shanghai, 200065, China.

**Keywords:** Exosomes, miR-139-3p, colorectal cancer, metastasis

## Abstract

**Objectives:** This study investigated plasma exosomal miRNA-139-3p as a blood-based biomarker for the early diagnosis and metastasis monitoring of colorectal cancer (CRC).

**Patients and Methods:** Exosome-rich fractions were isolated from the plasma of 80 CRC patients, and 23 controls using a kit method. We then used real-time polymerase chain reaction (RT-qPCR) to detect miR-139-3p levels in all subjects to evaluate expression levels and the predictive value of plasma exosomal miR-139-3p in CRC. We also collected clinicopathological data to explore correlations between abnormal miR-139-3p expression and clinicopathological parameters.

**Results:** When compared with healthy controls, exosomal miR-139-3p expression levels in CRC patients were significantly down-regulated. Furthermore, these expression levels were lower in metastatic colorectal cancer (mCRC) and submucosal patients. Receiver operating characteristic (ROC) curve analysis showed that exosomal miR-139-3p levels were differentiated between CRC patients and healthy controls, as well as between non-metastatic and metastatic patients.

**Conclusion:** Our findings show that decreased exosomal miR-139-3p expression levels in CRC patient plasma may act as a novel biomarker for the early diagnosis and metastasis monitoring in CRC.

## Introduction

Globally, CRC is a major factor in cancer mortality and morbidity 1. When initially diagnosed, approximately 21% of patients will have metastatic disease, and over 50% will develop metastases in the course of the disease 2. These factors make CRC one of the deadliest classes of cancer. Therefore, screening procedures are essential in reducing CRC morbidity and mortality. Currently, several CRC screening method exist: stool testing, carcinoembryonic antigen (CEA), colonoscopy, colonography, computed tomography (CT), and double-contrast barium enemas for detecting cancer and precancerous lesions 3. However, some of these methods are invasive, or have limited sensitivity and specificity. To address these clinical challenges, reliable and non-invasive diagnostic biomarker discovery is paramount.

Exosomes are nano-sized vesicles (30-150 nm in diameter), released into the extracellular environment by the fusion of multivesicular bodies with plasma membrane, in the blood 4. They are surrounded by a lipid bilayer, which carries a variety of biomolecules including, glycans, proteins, metabolites, lipids, DNA and RNA 5. Several studies have shown that exosomes express high levels of miRNAs, and that they contribute to chemo-resistance, immunomodulation and metastasis in various tumor types 6. Similarly, previous studies have indicated that cells actively secrete miRNAs into the extracellular environment via exosomes, and that these molecules function as biological messengers, communicating between cells 7-9. It is believed that exosomal miRNAs reflect some of the physiological changes and disease progression mechanisms in the human body. Moreover, the exosomal outer membrane prevents RNA degradation during *in vivo* circulation. Therefore, the discovery of effective biomarkers in exosome cargoes for early detection of cancer has aroused widespread interest in the scientific community.

In this study, we selected the plasma exosomal miRNA (miRNA-139-3p), identified by RNA sequencing. We investigated and validated this marker in 80 CRC patients, and 23 healthy controls, and evaluated its diagnostic potential by comparing clinical and pathological characteristics in these patients. Our data suggests that plasma exosomal miRNA-139-3p can be used as a complement to existing biomarker predictors to advance the differential diagnosis of CRC.

## Materials & Methods

### Patients & clinical samples

We collected peripheral blood samples from patients with CRC who underwent surgical resection at Tongji Hospital of Tongji University, from July 2016 to April 2018. All patients were confirmed by pathologist after operation. The ethics committee at Tongji Hospital approved this study, allowing us to obtain informed consent from each patient, prior to study commencement. All patients signed consent sheets. We finally recruited 80 colorectal cancer patients, and 23 healthy controls. Each blood sample was processed within 30 min of collection. All samples were centrifuged for 15 min at 3000×g at 4 °C, and then pumped and stored at -80 °C. Following the AJCC Cancer Staging Manual, (7th edition, 2009) 10, patients were classified into groups with or without metastasis. In addition, we collected relevant clinical data from patients including age, gender, depth of invasion, tumor location and lymph node metastasis.

### Plasma exosome isolation

Plasma exosomes were extracted by ExoQuick-TC^TM^ (SBI, Palo Alto, CA) kit method. After frozen plasma samples were equilibrated to room temperature, samples were centrifuged at 3000×g for 15 min to remove residual cells and cell debris. 250 µl supernatant was removed into a new tube, to which 63 µl ExoQuick reagent was added. The tube was thoroughly mixed and incubated at room temperature for 30 min. This was followed by centrifugation at 1500×g for 30 min, after which the supernatant was discarded. This step was repeated for another 5 min, after which the supernatant was discarded again. Finally, the remaining pellet was deemed as the exosome fraction.

### Transmission Electron Microscopy (TEM)

A 20 µl exosome aliquot suspension was placed onto a 200-mesh carbon coated copper grid for 2 min. After excess liquid was removed using filter paper, the grid was negatively stained with a 3% tungsten phosphate solution at room temperature for 3 min. The copper mesh was washed five times in double distilled water, and allowed to dry naturally at room temperature. The sample was observed and photographed under the transmission electron microscope (Thermo-Fischer, Waltham, MA, USA).

### Nanosight particle tracking analysis (NTA)

To identify the size distribution of isolated particles, a NanoSight LM10 system (Malvern Instruments Ltd. Malvern, UK) was used to size the diluted exosomes. Exosome concentration and particle size was determined by the NanoSight LM10 system software.

### Western blot analysis

Diluted exosomes were added to sodium dodecyl sulfate (SDS) buffer and boiled for western blot analysis. Primary antibodies for TSG101, CD63 and CD9 were obtained from Santa Cruz Biotechnology, Inc. (Texas, USA). Secondary antibodies were rabbit-anti-mouse from Dako (Carpinteria, CA, USA) or HRP-conjugated goat anti-rabbit antibodies from Santa Cruz Biotechnology, Inc. (Texas, USA).

### Total RNA extraction from plasma exosomes

Total RNA from exosomes was isolated using the miRNeasy Micro kit (QIAGEN, Germany) following the manufacturers' instructions. We determined RNA quality and quantity on the Agilent Bioanalyzer 2100 System (Agilent Technologies, CA, USA), following standard procedures.

### Quantitative real-time PCR

RT-qPCR was used to quantitatively assess miR-139-3p expression levels in plasma exosomes. First, apply exosome-miRNAs as template, using TagMan MicroRNA Reverse Transcription Kit (Takara, China) to reverse miRNA to cDNA. The systems are as follows: Total RNA50-500ng, dNTP mix (10mM) 0.5ul, MMLVReverse Transcriptase (200U/ul) 0.5ul, RT primers((10 µM) 1ul, 5×RT Buffer 5ul, RNase Inhibitor (20U/ul) 0.25ul, RNase free water up to 20ul. Polymerase Chain Reaction(PCR) conditions were: 25 °C 30 min, 42 °C 45min, 85 °C 10min to reverse miRNA to cDNA. Then apply cDNA as template, performed Realtime PCR using the Permix Ex TaqTM Kit (Takara, China) on an ABI 7300 (Applied Biosystems, Singapore) for quantification. The miR-139-3p F primer: 5'-TGGAGACGCGGCCCTGT-3'; the miR-139-3p R primer: 5'-TATGCTTGTTCTCGTCTCTGTGTC-3'. The total systems were: TaqMan Master Mix II 10.0ul, F+P primers1.0ul, cDNA+RNase-free water 9.0ul. Quantitative PCR conditions were: pre-denaturation at 95 °C, 10 min followed by 95 °C 12 s, 62 °C 40 s, totally 40 cycles. Use small nuclear RNA U6 (U6 snRNA) as internal reference, applying the formula: F =2^-ΔΔCT^ to calculate the fold-change in the expression of the above miRNAs.

### Statistical analysis

Data were represented by the mean ± standard deviation (SD), and compared using independent T tests, Wilcoxon tests and Kruskal-Wallis tests, as appropriate. We set the level of statistical significance at a two-sided to *P* < 0.05. Diagnostic values were assessed by receiver operating characteristic (ROC) curve analysis, and by computing the area under the curve (AUC), with confidence intervals (CI). We completed all analyses using SPSS (IBM Corp., Armonk, NY, USA) and GraphPad Prism 8 (GraphPad Software, Inc., La Jolla, CA, USA).

## Results

### Characterization of exosomes isolated from plasma

To validate kit efficiency in isolating exosomes, nanoparticles were tested using NTA and TEM, and three antibody markers for extracellular vesicles. TEM images revealed oval or bowl shaped microvesicles. NTA data showed that patient plasma exosome peak sizes were between 40 nm and 150 nm. Western blot analysis showed that exosomes were positive for the exosomal markers, Tsg101, CD63 and CD9. Therefore, exosome integrity and purification were confirmed.

### Plasma exosome-derived miR-139-3p levels at different CRC stages

**Figure 2:**
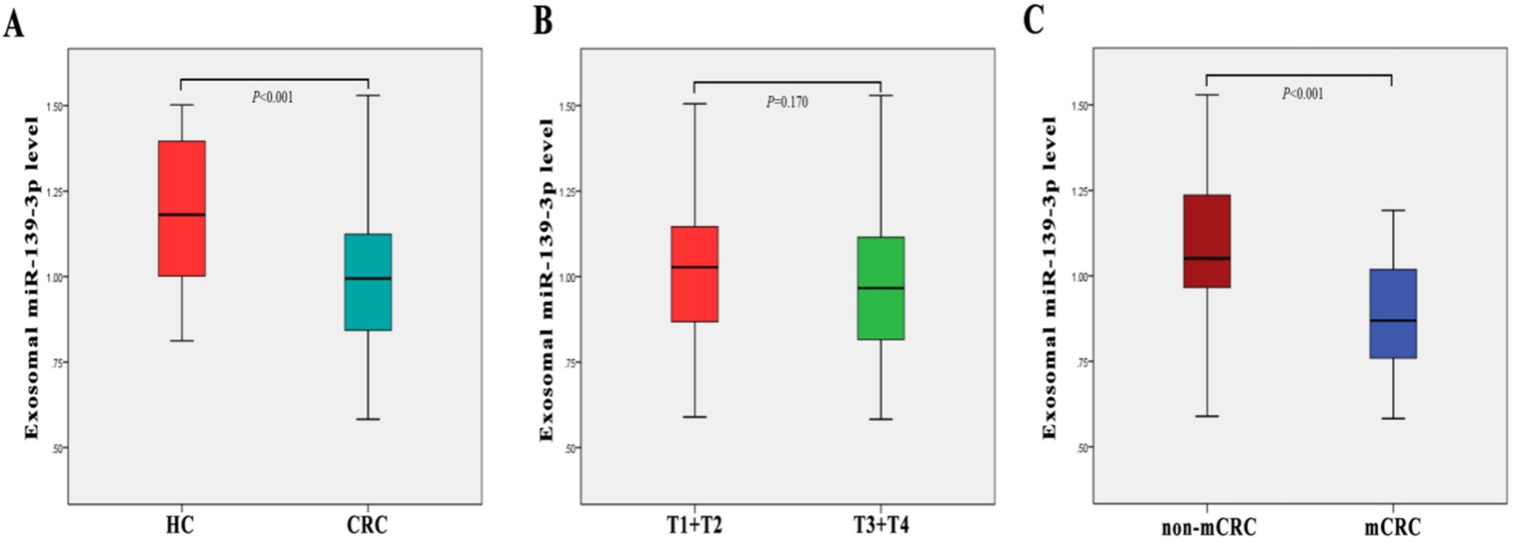
** Plasma exosome-derived miR-139-3p levels at different colorectal cancer stages.** (A) Exosomal miR-139-3p expression levels in plasma were significantly down-regulated in CRC patients when compared with healthy controls (B) No statistical differences were observed in plasma exosomal miR-139-3p levels between stages T1 + T2 and T3 + T4 patients. (C) Exosomal miR-139-3p expression levels in plasma were significantly down-regulated in metastatic colorectal cancer (mCRC) patients when compared with non-metastatic colorectal cancer (non-mCRC) patients.

The clinical stages of all 80 patients are shown in Table 1. We examined plasma exsomal miR-139-3p levels in CRC patients and healthy controls using RT-q-PCR. As shown (Fig. 2A), exosomal miR-139-3p expression levels in patient plasma were significantly down-regulated (*P* < 0.001), when compared with healthy controls. We also investigated plasma exsomal miR-139-3p levels in CRC patients at different disease stages. From Fig. 2C, exosomal miR-139-3p expression levels in patient plasma were significantly lower (*P* < 0.001) in mCRC patients than non-metastatic colorectal cancer (non-mCRC) patients. However, there were no significant differences in plasma exsomal miR-139-3p levels between T1 + T2 and T3 + T4 (*P* = 0.170) (Fig. 2B).

### Correlations between plasma exosomal miR-139-3p levels and CRC clinical parameters

**Figure 3:**
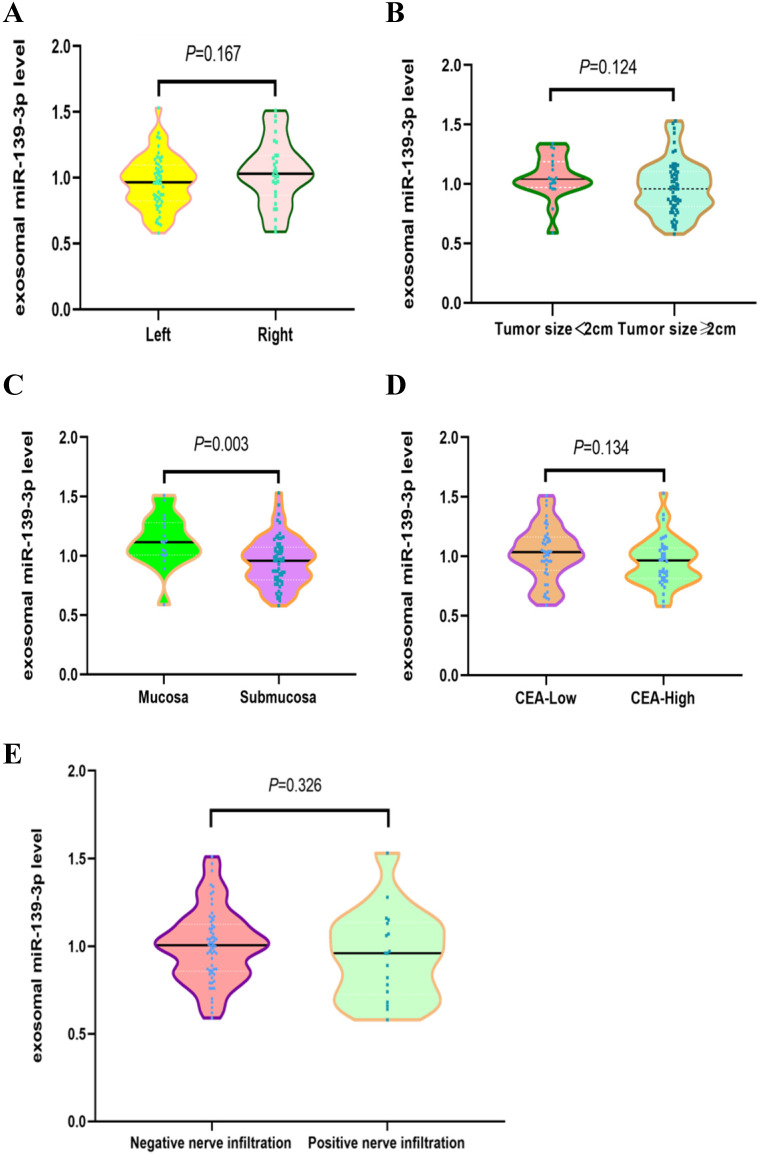
** Correlations between plasma exosomal miR-139-3p levels and CRC clinical parameters.** (A) No statistically significant differences were observed between left and right tumor locations (*P* = 0.167). (B) No statistically significant differences were found between tumor size; < 2 cm and ≥ 2 cm (*P* = 0.124). (C) Exosomal miR-139-3p expression levels in plasma were significantly down-regulated in the submucosal group when compared with the mucosal group (*P* = 0.003). (D) No statistical differences were observed in plasma exosomal miR-139-3p levels between low CEA and high CEA levels (*P* = 0.134). (E) No statistical differences were observed in plasma exosomal miR-139-3p levels between negative and positive nerve infiltration (*P* = 0.326).

We investigated potential correlations between plasma exosomal miR-139-3p levels and other clinical parameters. As shown (Fig. 3C), exosomal miR-139-3p expression levels in plasma were significantly down-regulated in the submucosal group when compared with the mucosal group (*P* = 0.003). However, no significant differences in plasma exsomal miR-139-3p levels were observed for patients with different tumor locations (Fig. 3A, Left *vs.* Right, *P* = 0.167), different tumor sizes (Fig. 3B, tumor size < 2 cm *vs.* tumor size ≥ 2 cm, *P* = 0.124), different CEA levels (Fig. 3D, low-CEA *vs.* high-CEA, *P* = 0.134), and different nerve infiltration (Fig. 3E, negative nerve infiltration *vs.* positive nerve infiltration, *P* = 0.326).

### Diagnostic value of each marker for colorectal cancer

**Figure 4:**
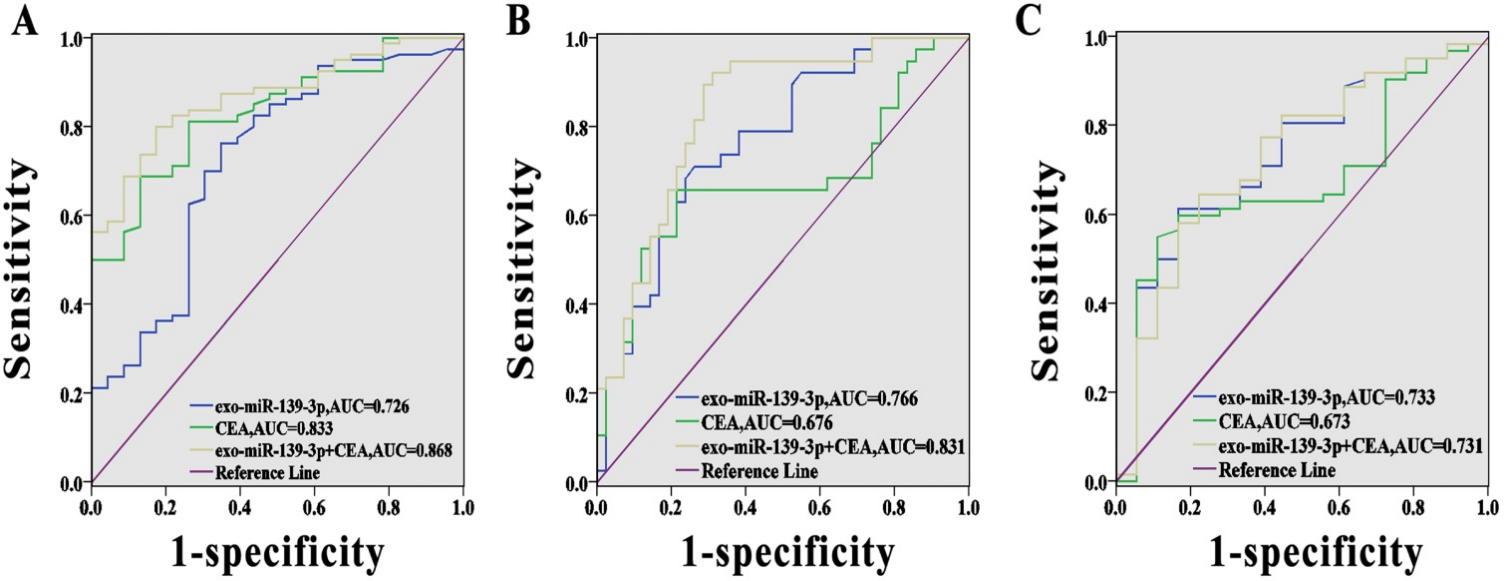
** Diagnostic power of each marker for CRC.** (A) The AUC for exosomal miR-139-3p, CEA, and combined indicators for CRC diagnosis were 0.726, 0.833 and 0.868, respectively. (B) The AUC for exosomal miR-139-3p, CEA and combined indicators for mCRC diagnosis were 0.766, 0.676 and 0.831, respectively. (C) The predictive value of exosomal miR-139-3p was better than CEA in terms of differentiating submucosal from mucosal patients.

We evaluated the predictive value of miR-139-3p using ROC curve analysis. As shown (Fig. 4A), the AUC (95% CI) for exosomal miR-139-3p and CEA, as CRC diagnostic biomarkers, were 0.726 (95% CI; 0.603-0.848) and 0.833 (95% CI; 0.751-0.915), respectively. Fig. 4B showed that the AUC (95% CI) for exosomal miR-139-3p and CEA for mCRC diagnoses was 0.766 (95% CI; 0.662-0.869) and 0.676 (95% CI; 0.552-0.800), respectively. However, when these two indicators were combined, the diagnostic performance was improved when compared to a single indicator (the AUC for the combined indicators for CRC diagnosis was 0.868 (95% CI; 0.797-0.938), and for combined indicators for mCRC diagnosis, the AUC was 0.831 (95% CI; 0.741-0.922). In addition, when we distinguished submucosal from mucosal patients, the AUC reached 0.733, which was superior to CEA.

## Discussion

As a clinical challenge, the early diagnosis and metastasis monitoring of cancer has received much attention. In recent years, small extracellular vesicles have provided the potential to act as minimally invasive diagnostic tools for asymptomatic cancer patients. Exosomes isolated from the blood contain multiple proteins and nucleic acid moieties, of which miRNA has become topical in biomarker research, due to its therapeutic potential and stability 11-13. miRNAs are protected from RNase activity thanks to several mechanisms: they are wrapped in membrane vesicles (microparticles, exosomes, and apoptotic bodies) and they are enclosed in lipoprotein complexes or associated with proteins 14-15. Recent studies have demonstrated that exosomal miRNAs may act as novel biomarkers for cancer diagnosis, e.g. exosomal miRNA-21 and miRNA-1246 in CRC 16-18. To date, this is the first study to examine the potential of plasma exosomal miRNA-139-3p in CRC diagnosis.

We determined that plasma exosomal miR-139-3p levels were down-regulated in CRC patients, when compared to healthy controls. Importantly, this observation distinguished CRC patients from the healthy control group. Consistent with a previous study, Liu *et al.,* 19 reported that miR-139-3p levels in colon cancer tissues were significantly lower than those in adjacent non-cancerous tissues, and that these lower levels were significantly correlated to poor overall patient survival. Also, we observed that exosomal miR-139-3p levels in the metastatic group were significantly down-regulated when compared with the non-metastatic group. Interestingly, when compared with the infiltrating mucosa group, exosomal miR-139-3p levels in the infiltrating submucosa were significantly down-regulated, processing AUC value of 0.733. Hence, these data suggest that plasma miR-139-3p levels may be negatively correlated with CRC disease progression, which may reflect disease severity. Notably, miR-139-3p was also down-regulated in other cancers including hepatocellular carcinoma and ovarian cancer 20-21. This accumulating evidence suggests that miR-139-3p may be a potential tumor suppressor. Finally, we evaluated the diagnostic efficacy of miR-139-3p and CEA combinations. The AUC for the combined CRC and mCRC diagnosis was 1 and 2, respectively, suggesting exosomal miR-139-3p may be a novel auxiliary diagnostic marker for CRC diagnosis.

Recent studies observed that exosomes, acting as messengers between tumors and stromal cells, transfer miRNAs from donor to adjacent cells 22. This interaction may induce reprogrammed gene expression in target cells and alter tumor growth, metastasis, and epithelial-mesenchymal transformation 23. For instance, Qi *et al.,* 24 demonstrated that exosomal miR-660-5p promotes tumor proliferation and viability by targeting KLF9, which leads to NSCLC progression. Moreover, Wang *et al.,* 25 reported that hypoxic tumor-derived exosomal miR-301a mediates M2 macrophage polarization via the PTEN/PI3Kγ signaling pathway to promote pancreatic cancer metastasis. However, the exosomal miR-139-3p mediated mechanism in CRC is still unclear.

To date, several studies have reported on the role of tissue or cell-derived miR-139-3p in other cancers, including hepatocellular carcinoma, ovarian cancer and glioma. Zou *et al.,* 20 showed that miR-139-3p inhibited the metastatic process in HCC, through the down-regulation of ANXA2R expression. Xue *et al.,* 21 observed that miR-139-3p inhibited ovarian cancer cell progression by inhibiting ELAVL1 expression. Furthermore, Shi *et al.,* 26 found that miR-139-3p was implicated in glioblastoma growth and metastasis inhibition by targeting NOB1, while another study found that miR-139-3p inhibited human glioma cell invasion, proliferation, and migration via the targeting of MDA-9/syntenin 27. These data suggest that miR-139-3p cancer regulatory mechanisms may involve multiple pathways, which require further investigation.

There were several limitations to this study. Firstly, there were no long-term clinical follow-up data for each CRC patient, thereby limiting the long term prognostic value of exosomal miR-139-3p. Secondly, due to the limited number of patients, the results must be confirmed with larger prospective studies in future. Thirdly, we did not explore the mechanism of exosomal miR-139-3p expression in CRC, however this is anticipated in future work.

## Conclusions

Plasma exosomal miR-139-3p was significantly down-regulated in CRC patients, and was associated with disease progression. Our study clearly indicated that exosomal miR-139-3p may be a promising blood-based biomarker for the early diagnosis and metastasis monitoring of CRC. As a non-invasive biomarker, this molecule has great potential as an auxiliary biomarker in the diagnosis of CRC.

## Figures and Tables

**Figure 1:**
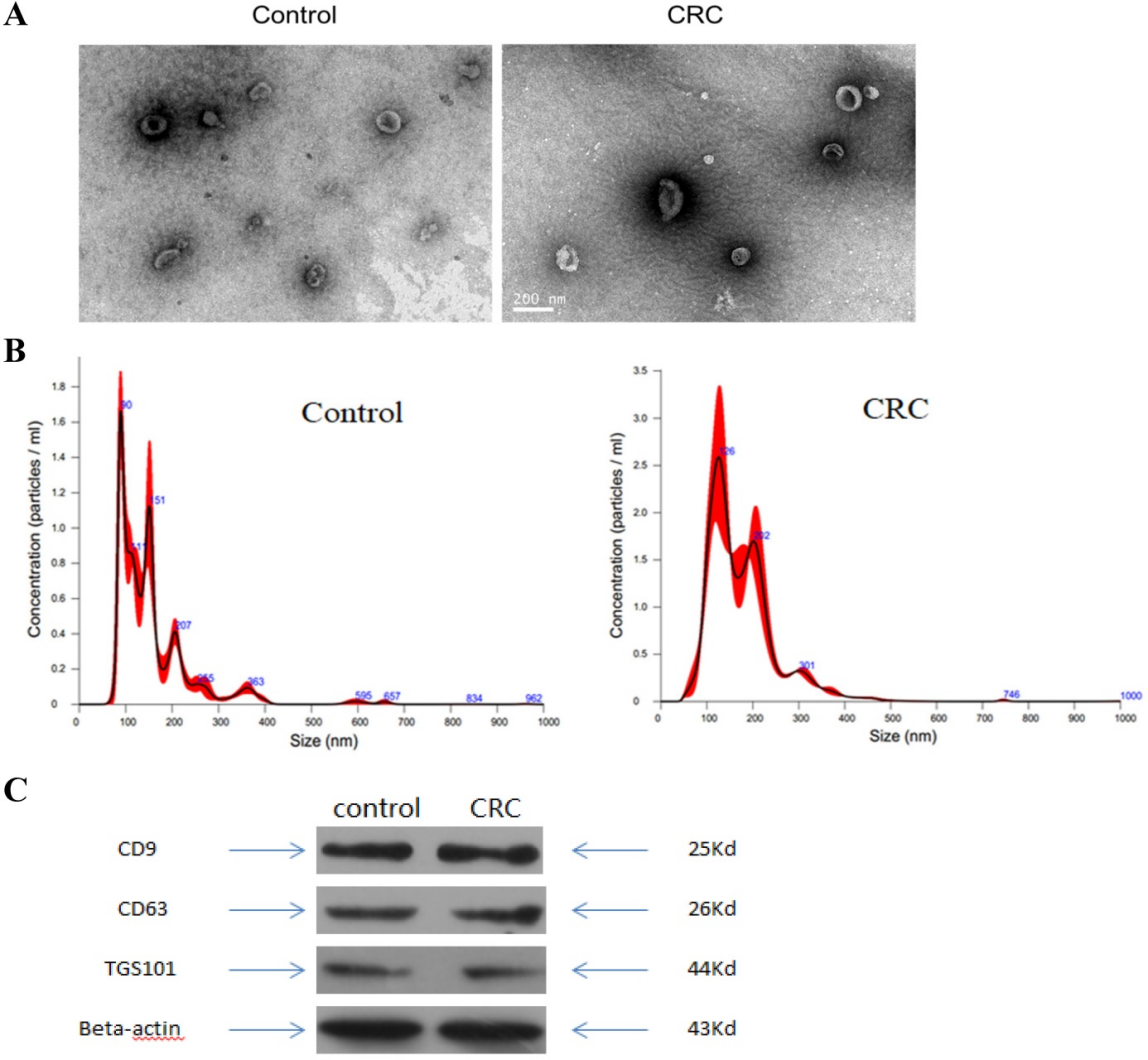
** Characterization of exosomes isolated from plasma.** (A) Transmission electron microscopy (TEM) showed the external features of the exosomes isolated from plasma. (B) Nanoparticle tracking analysis demonstrated the size distribution of the exosomes isolated from plasma. (C) Western blotting analysis of characteristic markers of extracellular vesicles, including TGS101, CD63 and CD9.

**Table 1:** Clinical characteristic of the patients

	Validation Cohort
Total number	80
**Age (years)**	
Median (min~max)	66 (41~93)
**Gender**	
Male	46 (57.5%)
Female	34 (42.5%)
**Tumor location**	
Left	52 (65.0%)
Right	28 (35.0%)
**Tumor size**	
<2cm	19 (23.8%)
≥2cm	61 (76.2%)
**Invasive depth**	
Mucosa	18 (22.5%)
Submucosa	62 (77.5%)
**Nerve infiltrate**	
+	18 (22.5%)
-	62 (77.5%)
**TNM Staging**	
T1+T2	26 (32.5%)
T3+T4	54 (67.5%)
N0	42 (52.5%)
N1	18 (22.5%)
N2	20 (25.0%)
M0	78 (97.5%)
M1	2 (2.5%)
**Metastasis (lymph nodes and distant)**	
Yes	38 (47.5%)
No	42 (52.5%)
**CEA**	
mean±SD	13.9±24.0

CEA, carcinoembryonic antigen.
